# Factors That Influence Exercise Among Adults With Arthritis in Three Activity Levels

**Published:** 2006-06-15

**Authors:** Cheryl Der Ananian, Sara Wilcox, Ruth Saunders, Ken Watkins, Alexandra Evans

**Affiliations:** Institute for Health Research and Policy, University of Illinois at Chicago. At the time this work was done, Dr Der Ananian was affiliated with the Department of Health Promotion, Education and Behavior, Norman J. Arnold School of Public Health, University of South Carolina, Columbia, SC; Department of Exercise Science, Norman J. Arnold School of Public Health, University of South Carolina, Columbia, SC; Department of Health Promotion, Education, and Behavior, Norman J. Arnold School of Public Health, University of South Carolina, Columbia, SC; Department of Health Promotion, Education, and Behavior, Norman J. Arnold School of Public Health, University of South Carolina, Columbia, SC; Department of Health Promotion, Education, and Behavior, Norman J. Arnold School of Public Health, University of South Carolina, Columbia, SC

## Abstract

**Introduction:**

Recent public health objectives emphasize the importance of exercise for reducing disability among people with arthritis. Despite the documented benefits of exercise, people with arthritis are less active than those without arthritis. The purpose of this study was to examine the factors that influence exercise participation among insufficiently active individuals with arthritis and to compare these factors with those identified by nonexercisers and regular exercisers with arthritis.

**Methods:**

Forty-six individuals with arthritis were recruited from various community-based organizations to participate in seven focus groups segmented by exercise status and education. Trained moderators led each discussion using a standard guide. All focus group discussions were transcribed verbatim and coded.

**Results:**

Pain was the most commonly mentioned barrier to exercise and limited exercise participation for nonexercisers and insufficiently active individuals. Paradoxically, insufficiently active individuals also identified exercise-related reductions in pain as a potential motivation for increasing exercise. Likewise, exercise-related reductions in pain were a motivation to continue exercising for the exerciser groups. Nonexercisers expressed that a reduction in pain was a possible outcome of exercise but were skeptical of its occurrence. Receiving tailored advice from a health care provider was consistently identified as an exercise enabler across the groups.

**Conclusion:**

Findings from this study indicate that potential strategies for increasing exercise participation include incorporating pain management strategies and coping skills into exercise interventions and ensuring that health care providers provide specific exercise advice to their patients with arthritis.

## Introduction

Recent public health initiatives emphasize the role of exercise in reducing arthritis-associated disability (1,2). Regular exercise is associated with numerous arthritis-specific benefits, including a delay in the onset of disability; improved physical functioning; enhanced functional independence; improved quality of life, aerobic capacity, and muscle strength; reduced pain; and a reduction in the risk of other chronic illnesses (1,3-12). Despite these benefits, more people with arthritis (31%) are sedentary than people without arthritis (26%) (13). Moreover, the prevalence of sedentary behavior among individuals with arthritis-related physical limitations is even higher (47%) (14).

A better understanding of the factors that influence exercise will improve the design of exercise intervention programs and strategies to refer, recruit, and market exercise programs to people with arthritis. Compared with the literature on the correlates of exercise in the general population (15), the current body of knowledge on the correlates of exercise for people with arthritis is limited. A recent review identified 36 studies that have included correlates of exercise; however, most of the studies were not designed specifically to examine correlates (16). 

Different variables might be associated with different phases or levels of exercise. For example, although perceived barriers may not show an association with exercise in an underactive sample, certain barriers may be important after an individual begins exercising, and other barriers may be relevant to maintaining an exercise routine. Given the high percentage of underactive individuals (45%) with arthritis (13), it is important to understand what factors may enable these individuals to increase their level of exercise and prevent them from becoming less active.

To date, only three qualitative studies have examined exercise and arthritis and, of these studies, two did not measure current exercise habits (17-19). Qualitative research can be particularly useful when studying populations that have received little study (20) because it explores the influences on exercise from the participants' perspectives without being constrained by the researcher's understanding of the related constructs. The purpose of this study was to examine factors that influence exercise participation among insufficiently active adults with arthritis using focus group methodology. Similarities and differences in the benefits, enablers, and barriers to exercise were compared between exercisers and insufficiently active individuals and between insufficiently active individuals and nonexercisers. Similarities and differences between exercisers and nonexercisers are reported elsewhere (21).

## Methods

### Design

A qualitative design was used for this study. The social–ecological model (22) informed the structure of the moderator's guide for the focus groups, and a grounded theory approach (20) was used to analyze the results of the focus groups.

Three focus groups were conducted among insufficiently active individuals with greater than a high school education. Our original intent was to conduct two groups with insufficiently active individuals; however, the sample size of one of the groups was too small, requiring us to conduct a third group. Results of these three focus groups were compared with two similar focus groups conducted with exercisers and two similar focus groups conducted with nonexercisers from a larger study (21). Groups were segmented by exercise status to create homogeneity and to help participants feel more comfortable and willing to talk openly (23). Thus, data from seven focus groups were available for analysis.

### Instrument

Facilitators used a moderator's guide premised on the social–ecological model (22). The moderator's guides for the exercisers and nonexercisers were developed and pilot tested as part of a larger study, and their development is described elsewhere (21). The guide used for the insufficiently active individuals (Appendix) was based on the moderators' guides for the exercisers and nonexercisers. Slight modifications were made to tailor the questions to insufficiently active individuals.

### Focus group procedures

Upon approval from the University of South Carolina Institutional Review Board, study personnel recruited participants through advertisements placed in local community-based organizations, newspapers, and radio stations. After providing verbal consent, interested participants were screened over the telephone, and eligible participants were invited to attend a focus group. Individuals aged 18 years and older with greater than a high school education and a self-report of a physician's diagnosis of any type of arthritis and who were classified as an exerciser, an insufficiently active individual, or a nonexerciser during screening were eligible to participate.

The focus groups were moderated by two women with training and experience in conducting focus groups. Each discussion lasted 75 to 90 minutes, and each participant received a $30 honorarium. All groups were audiotaped, and participants' comments were transcribed verbatim. Transcripts were reviewed for accuracy before analysis. 

### Analysis

All personnel involved in coding and analyses attended three training sessions to review principles of qualitative research and to acquire the skills necessary for using NVivo qualitative software (QSR International, Doncaster, Victoria, Australia). Initially, all research team members independently read the focus group transcripts. Together, the team generated a list of themes that were organized into a code book with definitions that served as the framework for coding.

Subsequently, two coders were assigned to code each of the groups. Each coder independently coded the transcript, and the pair met to review all codes and reach a consensus. Consensus codes for all focus groups were entered into NVivo. Coding was iterative, with new codes and their definitions added as needed. All coders were informed of new codes, and previously coded transcripts were recoded to reflect the changes.   

The unit of analysis was the focus group rather than the individual participant. In focus groups, participants often express agreement with one another by nodding their heads, and thus an analysis of simple frequency counts of themes is not a good indicator of the importance of a theme. Results are reported according to how many groups of exercisers, insufficiently active individuals, and nonexercisers expressed the theme.

### Additional measures

Participants reported their age, sex, race, educational attainment, income, and employment status. Participants also reported their arthritis type (based on a physician diagnosis) and duration in years.

A modified version of the 2001 Behavioral Risk Factor Surveillance System (BRFSS) physical activity module was administered during the telephone screening (24). The 2001 questions were designed to evaluate leisure-time moderate, vigorous, and strength-training activities, including lifestyle activities. However, for this study, the questions were modified to obtain information only on structured exercise, with lifestyle-related activities excluded. Participants were asked to report the type, frequency, and duration of their moderate- and vigorous-intensity structured exercise and strengthening exercises.

Based on their responses, participants were classified into one of three groups. Exercisers reported participation in moderate exercise on 3 or more days per week for at least 30 minutes per day *or* in strength training or vigorous activities on 3 or more days per week for at least 20 minutes per day. Participating in this level of exercise has been shown to yield health benefits among people with arthritis (25-27). Nonexercisers reported exercising 1 day per week or less. Participants who reported exercising 10 minutes or less on 2 days per week were also considered nonexercisers. Insufficiently active individuals were participants who did not meet the criteria for either the exercise or nonexerciser categories.

### Participants

Participants were a volunteer sample of 46 men and women from the greater Columbia, SC, area (Lexington and Richland counties). Thirty-one of the participants were originally recruited to participate in a larger qualitative study examining the factors that influence exercise among nonexercisers and exercisers with arthritis (recruited from May 2003–March 2004). The remaining 15 were recruited to participate in this study (recruited from June 2004–March 2005).

Table 1 provides an overview of the sociodemographic characteristics of the sample, stratified by exercise status. Exercisers, insufficiently active individuals, and nonexercisers did not differ in age, sex, race, or income level, and the sample was fairly evenly distributed across these variables.

## Results

### Barriers to exercise

Four broad categories of barriers to exercise emerged: physical, psychological, social, and environmental. Table 2 lists the themes that emerged for the physical and psychological barriers to exercise. Table 3 illustrates the themes for the social and environmental barriers to exercise. Because findings for exercisers and nonexercisers have been reported elsewhere (21), emphasis is placed on comparisons between these two groups and the insufficiently active groups.

**Physical**

**Pain.** Pain was the most frequently stated barrier to exercise among all participants. Pain during or after exercise was most commonly expressed by the exercisers. Exercisers often described modifying their exercise routine to accommodate their illness and working through their pain to maintain their level of exercise. Insufficiently active individuals and nonexercisers expressed experiencing pain before, during, and after exercise. A subtle distinction existed between nonexercisers and insufficiently active individuals. Whereas insufficiently active individuals described trying to exercise despite their pain, nonexercisers tended to stop exercising altogether. Both groups were skeptical that more exercise was possible.

**Mobility.** Mobility limitations emerged as a theme within all the insufficiently active groups but were only mentioned in one group each for the exerciser and nonexerciser groups. Issues described by insufficiently active individuals included limitations in their ability to perform activities of daily living and to walk or "get around." When exercisers and nonexercisers discussed mobility issues, their descriptions were vague and included statements such as "my knees don't work" and "you just can't move them [knees]." 

**Comorbidities. **Other illnesses were considered barriers to exercise. Cardiovascular disease was consistently mentioned as a barrier by exercisers and insufficiently active individuals. It was also mentioned as a barrier among nonexercisers, albeit less frequently. Additionally, diabetes and intervertebral disk issues were described as limiting exercise.

**Fatigue. **Fatigue was most commonly mentioned by the nonexercisers, with participants indicating that fatigue from their disease, the medications used to manage arthritis, or both prevented exercise. One group of insufficiently active individuals described "chronic fatigue" as a barrier to exercise and expressed the importance of rest or taking "down time" to get around it. Fatigue did not emerge as a theme among the exercisers.

**Psychological**

**Attitudes and beliefs.** Participants in all groups identified attitudes and beliefs that limited exercise; however, the barriers were qualitatively different among groups. Insufficiently active individuals consistently expressed the belief that arthritis limited their ability to exercise, and they expressed a lack of understanding about which exercises were safe or appropriate. They also believed that exercise was not reducing their pain level or affecting their symptoms as they thought it would. Less frequently, time constraints and not making exercise a priority were described as barriers by insufficiently active individuals. In contrast, the majority of exercisers expressed laziness, a lack of enjoyment, or time constraints as their main barriers. The main barrier expressed by the nonexercisers was similar to that expressed by the insufficiently active individuals: uncertainty about the types of exercise they should be doing. Nonexercisers also mentioned a lack of motivation for exercise and time constraints as barriers.

**Perceived negative outcomes.** All three groups believed that engaging in exercise would result in negative consequences (e.g., pain, stiffness). However, a subtle distinction occurred between the groups. Insufficiently active individuals consistently expressed that there was "a price to pay" for engaging in exercise, even when doing exercises they felt were appropriate. This price was typically described as increased pain or "not being able to move the next day." In contrast, exercisers acknowledged there could be negative consequences of exercise but ascribed these to "pushing yourself too far." Exercisers' descriptions emphasized the importance of knowing one's limits. Similar to the insufficiently active individuals, nonexercisers associated exercise with pain afterwards, and this fear prevented them from engaging in exercise.

**Social and environmental**

**Insufficient advice from health care providers.** Inadequate advice from a health care provider was a salient theme for the insufficiently active individuals. They indicated that advice from their physicians often lacked concrete details on the type, frequency, or intensity of exercise that is appropriate for people with arthritis. Many described receiving only a pamphlet or handout with exercise instructions or receiving vague instructions such as "yoga would be really good for you" without referral to an appropriate facility. Several participants also described not receiving any information about exercise from their health care provider. This theme was not frequently mentioned in the groups with nonexercisers or exercisers.

**Competing roles and responsibilities. **Participants in all three groups of insufficiently active individuals described their work-related or family responsibilities as barriers to exercise. This theme did not emerge for exercisers or nonexercisers.

**Natural environment.** The only natural environmental barrier that emerged among both the exercisers and insufficiently active individuals was weather. Cold or damp weather was described as exacerbating arthritis and decreasing the ability to exercise.

**Lack of exercise programs.** The lack of availability of arthritis-specific exercise facilities or programs emerged as a barrier for exercisers, insufficiently active individuals, and nonexercisers alike. Participants in all groups stated that arthritis-specific exercise programs were unavailable within the community. Of special concern was the lack of water aerobic programs in the community. Participants also described the need for instructors who understood arthritis and were knowledgeable about exercise for people with arthritis.

### Benefits and enablers of exercise

All participants were asked to describe 1) the advantages of exercise for people with arthritis, 2) the one outcome that made exercise worth doing, and 3) what motivated them to exercise. From these questions, two categories of benefits (physical and psychological) and four categories of enablers (physical, psychological, social, and environmental) emerged. The attainment of a benefit was typically viewed as an exercise enabler, and for this reason, benefits and enablers are discussed together. Table 4 contains an overview of the physical and psychological enablers of exercise; Table 5 illustrates the social and environmental enablers of exercise.

**Physical**

Three main types of physical benefits and enablers emerged: symptom management, improved mobility and physical functioning, and weight loss.

**Symptom management.** The most commonly mentioned benefits of exercise by exercisers, insufficiently active individuals, and nonexercisers alike were reductions in pain and stiffness. Exercisers and insufficiently active individuals described how exercise had reduced their pain levels. However, insufficiently active individuals were more likely to describe increased pain during exercise followed by a reduction in stiffness or pain afterward. Although exercise did not completely alleviate pain, both exercisers and insufficiently active individuals expressed that pain had become more tolerable through exercise. Reductions in pain before exercise, during exercise, or both were described as key factors that would enable insufficiently active individuals to increase their exercise. In contrast to insufficiently active individuals, nonexercisers recognized that pain reduction was a potential outcome of exercise but expressed skepticism. Nonexercisers indicated that *if* they could achieve pain reduction through exercise, they would be motivated to exercise.

**Improved mobility.** Exercisers indicated that improved mobility, including improved ability to perform activities of daily living, improved strength and flexibility, and improved ability to get around, were beneficial outcomes of exercise. Similarly, insufficiently active individuals recognized that *when* they exercised, it was easier to get around. In contrast, the nonexercisers acknowledged improved mobility as a potential outcome of exercise but expressed skepticism that it could occur. Similar to the findings on pain, they illustrated this skepticism by starting their statements with "if," "it might," or "I guess."

**Psychological**

**Independence.** Only exercisers identified maintaining their independence as a beneficial outcome of exercise that motivated them to continue exercising. This sentiment was captured in phrases such as "enables me to continue to do things," "I'm not an invalid," and "it's made me self-sufficient."

**Feeling better.** Nonexercisers, insufficiently active individuals, and exercisers identified "feeling better" as a beneficial outcome of exercise. This notion was illustrated in phrases such as "feeling better," "lifts your spirits," and "improves your mood." However, nonexercisers tended to describe their experiences with exercise before they had arthritis rather than their current experiences. Insufficiently active individuals and exercisers generally expressed that exercise currently helped them to feel better.

**Reducing stress.** Participants in two of three focus groups with insufficiently active individuals stated that exercise was a way to "relieve stress" or to "relax." This theme was not discussed in either group of exercisers and was only mentioned once among the nonexercisers.

**Making exercise a priority.** Participants in two focus groups with exercisers and one group of insufficiently active individuals stated that "making exercise a priority" enabled them to exercise. Consistent with self-regulation principles, participants described that participating in exercise involved "making time to exercise," "making exercise a personal goal," and "putting oneself first."

**Self-motivation.** Exercisers consistently described themselves as being "internally motivated" to exercise or as "self-starters." Similarly, participants in one of the three focus groups with insufficiently active individuals questioned how they could become self-motivated for exercise. This theme did not emerge among the nonexercisers.

**Social and environmental**

**Social support enablers.** Two types of social enablers emerged as themes: general social support for exercise and having someone with whom to exercise. The insufficiently active groups and the exerciser groups both described receiving support and encouragement to exercise from family members and friends. Both indicated that significant others reinforced the importance of exercise. Many of the insufficiently active individuals also indicated that having a group of similar individuals with whom they could exercise would provide an additional level of encouragement and support. Participants in the nonexerciser groups echoed this theme by stating that an exercise group comprised of similar individuals would increase their confidence to exercise.

Having someone with whom to exercise emerged as an enabler for both exercisers and insufficiently active individuals. Exercisers stated that exercising with others provided an opportunity for social interaction and was more enjoyable than exercising alone. Insufficiently active individuals stated that they were more "apt to go and do it" when they had someone with whom to exercise. In contrast to the exercisers and insufficiently active individuals, nonexercisers tended to state that *if* they had someone with whom to exercise, then they would be more likely to go.

**Health care provider advice.** A key enabler of exercise for participants in exerciser, insufficiently active, and nonexerciser groups was receiving a health care provider's advice. Exercisers often described receiving advice as what gave them the initiative to start an exercise program or how they learned about which types of exercise are most appropriate. The insufficiently active individuals agreed with the exercisers but also emphasized the importance of receiving detailed advice about the type, intensity, duration, and frequency of exercise and the appropriate places to go to exercise. Most nonexercisers reported receiving exercise advice from physicians, but their descriptions of the advice were vague and frequently referred to past advice.

**Access to exercise programs.** Among the exerciser groups, the presence of water aerobics at an exercise facility was described as a motivation to exercise. Among the insufficiently active individuals and the nonexercisers, the presence of knowledgeable instructors and the availability of individualized, tailored programs emerged as a theme. Key components of these tailored programs included having similar individuals with whom to exercise and the presence of instructors with firsthand knowledge of arthritis. Water aerobics was also mentioned by insufficiently active individuals, although less frequently than by the exercisers.

## Discussion

Consistent with the social ecological model, the findings of this study indicate that there are several physical, psychological, social, and environmental factors that influence exercise participation (22). Our findings were also consistent with the transtheoretical model (28,29): many factors varied by exercise level and provide important implications for designing intervention programs.

The primary barrier to exercise among exercisers, insufficiently active individuals, and nonexercisers was pain. However, exercisers tended to express that they had the coping skills or the knowledge necessary to alter their exercise routine to continue their exercise. In contrast, insufficiently active individuals felt that they could not do any more exercise because of their pain, did not know which exercises were safe or appropriate to do, and appeared to not have the skills necessary to modify their exercise routines. Likewise, nonexercisers seemed to lack the knowledge and skills necessary to tailor their exercise behaviors to manage their pain.

These findings have important implications for tailoring programs and suggest that incorporating pain management skills into an exercise program and teaching people how to modify their exercise routines according to their symptoms would be beneficial for nonexercisers and insufficiently active individuals. Consistent with this finding, pain management and exercise have been identified as the most important and beneficial aspects of arthritis self-management programs (30). The importance of teaching exercise modification skills and pain management is further emphasized by the paradoxical findings on pain. A reduction in pain was the most commonly cited beneficial outcome, and for the insufficiently active individuals, reduced pain was the most important motivating factor.

Health care providers' advice to exercise was important to all of the participants. However, insufficiently active individuals more commonly expressed the need for detailed advice on the types, frequency, duration, and intensity of exercise and for physician referrals on appropriate places to go. Increasing physicians' knowledge and self-efficacy for exercise counseling may be an important strategy for increasing exercise participation (18). Likewise, determining best-practice strategies for incorporating exercise counseling into doctors' visits, including identifying the type and intensity of counseling needed, is important for enhancing exercise participation, especially among those who are not regularly active. Recent evidence indicates that less than half of all adults with arthritis have ever received advice to exercise (31), yet physician-based counseling efforts that include written material and behavioral strategies have been shown to be effective at increasing physical activity (32).

Consistent with strategies suggested in the National Arthritis Plan (1), having an arthritis-specific exercise program was identified as an enabler of exercise, particularly among the nonexercisers and the insufficiently active individuals. Having instructors who are knowledgeable about both exercise and arthritis was viewed as important by participants in all groups. Also important was having instructors with firsthand knowledge of arthritis. Additionally, insufficiently active individuals and nonexercisers both expressed a desire to have a group of similar others with whom to exercise. These findings emphasize the need to expand community-based programs to include programs tailored to people with arthritis (33). The use of lay people with arthritis who have been trained to deliver exercise programs may be an important component of these community-based programs.

There were limitations to this study. The number of focus groups included was relatively small, although the study had a fairly large sample size relative to other qualitative studies conducted in this area (17-19). Additionally, consistent with qualitative research methods, a purposive sample was recruited for this study. This sample was predominantly white, female, and of higher socioeconomic status (as indicated by education), and results may not be generalizable. Participants were from only one metropolitan area in South Carolina, and limited access to arthritis-specific programs in this area may not be representative of access in other places. Participants also provided a self-report of exercise behavior and of arthritis type, and self-reports are subject to biases. Finally, focus groups were conducted among individuals with an array of rheumatic diseases and were not segmented by disease type. Therefore, this study could not provide information about how disease types may influence factors related to exercise. Future research in this area is warranted.

Despite these potential limitations, this study provided valuable information on how exercisers and insufficiently active individuals and nonexercisers and insufficiently active individuals differ on the factors that influence activity. It also provides important insights into intervention strategies (e.g., health care provider counseling, use of people with arthritis as exercise instructors) and marketing strategies that could be used to enhance exercise participation.

## Figures and Tables

**Table 1:** Sociodemographic and Disease-related Characteristics of Nonexercisers, Insufficiently Active Individuals, and Exercisers With Arthritis (N = 46)

**Characteristic**	**Nonexercisers (n = 15)**	**Insufficiently Active Individuals (n = 15)**	**Exercisers (n = 16)**
**Age, y, mean (SD)**	56.7 (10.4)	52.4 (9.6)	56.9 (14.5)
**Education, y, mean (SD)**	15.0 (1.6)	14.9 (1.8)	16.3 (2.3)
**Sex, no. (%)**			
Female	14 (93.3)	12 (80.0)	14 (87.5)
Male	1 (6.7)	3 (20.0)	2 (12.5)
**Race, no. (%)**				
White	8 (53.3)	11 (73.3)	14 (87.5)
Other^a^	7 (46.7)	4 (26.7)	2 (12.5)
**Annual income, no. (%)**			
≤$29,999	5 (41.7)	5 (35.7)	3 (20.0)
$30,000-$59,999	4 (33.3)	5 (35.7)	5 (33.3)
≥$60,000	3 (25.0)	4 (28.6)	7 (46.7)
**Body mass index, kg/m^2^, mean (SD)**	28.7 (5.5)^b^	31.0 (8.0)	29.6 (6.2)
**Type of arthritis, no. (%)^c^**			
Osteoarthritis	10 (71.5)	8 (61.5)	8 (50.0)
Rheumatoid arthritis	4 (28.6)	3 (23.1)	6 (37.5)
Fibromyalgia	8 (57.1)	5 (38.5)	5 (31.2)
Gout	0	1 (6.7)	0
Other	2 (14.3)	1 (6.7)	4 (25.0)
**Minutes of physical activity per week, mean (SD)**	16.3 (25.8)	58.7 (31.7)	241.9 (130.0)

a Other includes a self-reported race of African American, Hispanic, American Indian, or other.

b n = 14.

c Participant could report more than one type of arthritis; therefore, totals may exceed 100%

**Table 2:** Physical and Psychological Barriers to Exercise With Illustrative Comments Expressed by Focus Group (N = 7) Participants, by Exercise Level

**Theme Identified**	**Exercisers (n = 2)**			**Insufficiently Active Individuals (n = 3)**		**Nonexercisers (n = 2)**			

	**No. of Groups **	**Illustrative Comment**		**No. of Groups **	**Illustrative Comment**	**No. of Groups **		**Illustrative Comment**	
**Physical barriers**									
Pain									
Pain before exercise	1		"The cycle of pain keeps me from being consistent with what I want to do."	3	"I do what I can exercisewise. You know, I have a lot of days where it hurts to do anything. And everything just seems to aggravate it."	2		"Sometimes if it's a bad knee day, . . . well, if I have a bad knee day or a bad back day, I can't do anything."	
Pain during exercise	2		". . . but I can walk a little while and sit down on one of those benches and rest and let the worst of the pain calm down, and I can walk a little bit more to another bench. And I make it that way."	3	"But the more I walk, the more I walk, it seems like it makes the pain worse."	2		"Right, my trouble is really walking. You know it hurts to walk on my legs."	
Pain after exercise	2		"I walk, but I hurt so much afterwards."	3	"But if I'm just out doing an exercise that I do, walking swiftly with some hand weights, there's a price to pay afterwards. Usually the night of and the next day."	2		"I may be able to go walk with you, play tennis one day, and do whatever you want to do. I could do it with you, but for the rest of the week I'm bedridden."	
Mobility	1		"Since I have the arthritis in the lower part of my body,. . . I get down and I can't get up, or I'd be up and I can't get down."	3	"And the time leading up to being diagnosed it was hard to walk around the block."	1		". . . I like to walk but sometimes my knees or my back or whatever does not work."	
Comorbidity	2		"See, when you're diabetic you can't do those things, whether you want to or not."	2	"I can't do any load-bearing exercises like put the bar across and squatting because of my disk problems, but I do just about everything else with free weights."	1		"I've never been able to just be on a routine of exercise because of my asthma. I've had asthma my whole life, and it really has hindered me from being active."	
Fatigue	0		-----	1	"I have to rest to get around it. I don't know if that's really what you are asking, but I have to take down time a lot."	2		"But just for a moment you think, 'Boy, I could take a walk or I could ride the bicycle if I wanted to.' And it's gone just like that. By the time I would get my regular bike out, I'd be worn out and I'd leave it sitting."	
**Psychological barriers**									
Attitudes and beliefs	2		"For me plain laziness. I have plenty of time. I work at home. There is no excuse. And I can come up with 50 a day. It's just that I don't want to do it. That's the biggest barrier for me."	3	"I don't know if I believe it, but my doctor has assured me that if I did more walking that it would help with the discomfort, but I don't believe that. It has not proven true as of yet."		2		"My doctor has recommended swimming aerobics or water aerobics, and I understand there's a good class. My problem is that I have not done any of these things. It seems like just getting through the workday is about all you can handle."
Perceived negative outcomes	2		"So you really just have to know yourself, I think. You can push but you can also push too hard."	2	"Any time I do it, exercise, there's a price to pay at the end."		2		"Sometimes I've done things and I think, 'Oh man, I shouldn't have done that.' But what do I do instead?"

**Table 3:** Social and Environmental Barriers To Exercise With Illustrative Comments Expressed by Focus Group (N = 7) Participants, by Exercise Level

**Theme Identified**	**Exercisers (n = 2)**			**Insufficiently Active Individuals (n = 3)**			**Nonexercisers (n = 2)**	

	**No. of Groups **	**Illustrative Comment**		**No. of Groups **	**Illustrative Comment**		**No. of Groups **	**Illustrative Comment**
**Social barriers**								
Insufficient advice from health care provider	1		"And I don't know if it's here or I suspect it's probably nationwide that they don't tell you that. [The doctors], they're busy trying to calm the pain down and not maybe helping you as much with the exercise."	3		"And the only information I've gotten has just been minimal things from the doctors like, 'Yoga would probably be really good for you' or 'work on stretching the lower back.' You know, just minimal things."	1	"I will say that there is very little information being passed around that'll tell you you can go here or you can go there. It's sort of like a word of mouth thing."
Competing roles and responsibilities	0		-----	2		"My barrier is that fact that I work 3 days a week and I have two kids in the first grade and, you know, I try to do everything while they're at school so that when they're home, I can be like a mom. And you know I've got 8 million things to do and that is a big barrier to exercising."	1	"And it's all I can do to get up, go to work and take care of my family."
**Environmental barriers**								
Natural environment and weather	1		"I was walking since January. I'd take a break at work and two 15-minute times during the day, and I was doing okay until it got really hot. Now I can't do it outside because it's too warm."	1		"When the weather's bad and it's raining, you're aching and steps are almost impossible to go up."	1	"I'd been riding my regular bicycle and the weather had been so rainy and all."
Exercise programs	2		"They have some exercise classes. They aren't specifically for arthritis. They are exercise in general, but I am talking about for specifically arthritis self-help or fibromyalgia self-help or whatever."	3		". . . None that I know of. You hear all this about the gyms, . . . but, like he said, they are for healthy people, people that are not in pain that I know of. You never hear them say, 'Well, we have exercises for people with this problem or that problem.'"	2	"And then like the water aerobics. I love the water aerobics, but I live next to [town name]. . . and that's like a 40-minute drive."

**Table 4:** Physical and Psychological Benefits and Enablers of Exercise With Illustrative Comments Expressed by Focus Group (N = 7) Participants, by Exercise Level

**Theme Identified**	**Exercisers (n = 2)**			**Insufficiently Active Individuals (n = 3)**		**Nonexercisers (n = 2)**		

	**No. of Groups**	**Illustrative Comment**		**No. of Groups **	**Illustrative Comment **	**No. of Groups **		**Illustrative Comment**
**Physical benefits and enablers**								
Symptom management								
Reduced pain	2		". . . And mine was sheer pain. It was like once I learned that if I did something that stopped the pain a little bit. . . . Once I learned that I could do something that made a difference, it was like, well, let's do it."	3	"I didn't have near as much pain when I exercised. It's the pain I can't take."	2	"Knowing that those bad repercussions are not going to be there when you get through. Sure, let's go run a race."	
Reduced stiffness	2		"Well you start to loosen up a little bit. The stiffness seems to dissipate, you know, after, you know, moving, being mobile when I'm dancing or walking or whatever. I can tell a difference."	3	"To me what helps me most is exercise. I try to exercise three times a week, and I find that when I don't do it, I'm really stiff and painful. So, exercise and just actually moving around keeps me good."	1	"Because I agree that exercise does help arthritis because if you don't, you stiffen up."	
Mobility	1		"Well, I am sure I would be an invalid if I had not continued this exercise through all this time. I mean I may miss a time here or there or whatever, but I go right back."	3	"I'm sure everybody has heard the statement 'use it or lose it.' There are a lot of people that have testified to that. And I find that the more I move around, even though there is pain, it pays off in the long run."	1	"I'm [name] and possibly more mobility."	
**Psychological benefits and enablers**								
Feeling better	2		"I do it because I need to and I'm happy after. I feel good after. I just don't want to do it."	3	"I think exercise really lifts me up a bit. You know, it makes me feel better if I am not already hurting.	2	"I look forward to getting with the group. It was fun and I did get to feeling better. Now, how can I get back to it?"	
Making exercise a priority	2		"I make the time. It's just helped me so much that I make time."	1	"One of the things that I've learned to get around some of the barriers [to exercise]. . . . I've learned that I've got to take care of me first, you know. In spite of everything else that is going on, I've got to take care of me."	0	-----	
Self-motivation	2		"I am a self-starter. It's nice that my family and my friends support me, but I think I would do it whether they did or not."	1	"My family tells me you need to exercise, go exercise, and they really pushed me to exercise. And that's not really why I do it. I do it for myself."	0	-----	

**Table 5:** Social and Environmental Enablers to Exercise With Illustrative Comments Expressed by Focus Group (N = 7) Participants, by Exercise Level

**Theme Identified**	**Exercisers (n = 2)**			**Insufficiently Active Individuals (n = 3)**		**Nonexercisers (n = 2)**		

	**No. of Groups **		**Illustrative Comment**	**No. of Groups **	**Illustrative Comment **	**No. of Groups **		**Illustrative Comment**
**Social enablers**								
Health care provider advice	2		"The doctor was the motivating force, but he didn't know that much about it. He told me, you know, 'Here are three places to check out.'"	3	"I heard about the water aerobics when my doctor told me about it. And it has helped me to do other things just by the water aerobics."	2	"My doctor told me, he says it may mean your life if you exercise. Now I think that would be enough motivation. I'm still not doing it. It bugs me that I am such a failure at that."	
Social support	2		"I think it's very important for family, for friends to support you. To understand that there is something wrong with you, but they can encourage you."	3	"I've gotten most of my information from either a doctor or other people that have the same type of disease have given me a few ideas of how to exercise."	2	"So anyway, I have a daughter who's very supportive and she is just right there with me all the way."	
Someone to exercise with	2		"Well, I like being with the other people and exercising with them. . . . There's a social support there."	3	"And I've seen ladies a lot older than me in that aerobics class. Some of them are 80 and 90 years old, can't even walk outside of the water, you know. Being in the water they can do it. Seeing it, that's a big motivation."	2	"I think that an exercise program for those like us, we're a society by ourselves . . . it would be a wonderful thing."	
**Environmental enabler**								
Exercise programs	2	"They had water aerobics. . . . And now they have 3 classes. . . . And a lot of doctors are sending patients there particularly."		2	"I would feel more comfortable if this person [instructor] could relate to me saying, 'Well, I have had arthritis. I know what you're going through. I know what the pain is like.'"	2	"You'd really have to have a personal trainer who was trained to tailor [the program], which would be a wonderful thing."	

## Appendix. Main Questions From the Moderator's Guide for Focus Groups With Insufficiently Active Individuals With Arthritis

(Bolded questions were added to the moderator's guide for insufficiently active individuals.)

1. Let's go around the room and please introduce yourself and briefly tell us what you think is the ONE key ingredient for managing your arthritis successfully. 
2. Tell me the one most important way that your arthritis has affected you and the things you are able to do.
3. What types of exercises do you do now? Tell me about what you did in the last week.
4. Now I'd like you to think about the exercises you did before you developed or were diagnosed with arthritis. How do your previous experiences with exercise compare to the exercises you do now? In other words, how has your exercise changed as a result of your arthritis?
5. For those of you in the group who started to exercise since developing arthritis, what led or motivated you to *start* exercising?
6. What advantages or benefits, if any, do you think you might obtain from increasing your current level of exercise?
7. What would it take for you to increase your current level of exercise? That is, to exercise more often or for a longer time when you do exercise?
8. What is the one outcome or one thing that exercise does for you that makes it worth doing? 
9. What gets in the way of exercising for you? That is, what are your major barriers?
10. What do people who are important to you — like your family and friends — think about whether or not you should exercise?
11. What types of exercise programs or information about exercise are available for people with arthritis in your community?

I've been asking you a lot about your experiences with exercise. Now I'd like you to think about yourself but also think more broadly about other people with arthritis as you answer the next two questions. 

1. First, what are the advantages or benefits of exercise for people with arthritis?
2. Second, what are the disadvantages of exercise for people with arthritis?  These could include negative outcomes or results of exercise, costs, or bad things that might happen.
3. What did your doctor or health care provider tell you about exercise after you were diagnosed with arthritis?
4. Finally, is there anything else about arthritis and exercise that I did not ask you but would be useful for me to know?
